# Mental, neurologic, and substance use (MNS) disorders among street homeless people in Ethiopia

**DOI:** 10.1186/s12991-017-0163-1

**Published:** 2017-11-16

**Authors:** Getinet Ayano, Dawit Assefa, Kibrom Haile, Asrat Chaka, Haddish Solomon, Petros Hagos, Zegeye Yohannis, Kelemua Haile, Lulu Bekana, Melkamu Agidew, Seife Demise, Belachew Tsegaye, Melat Solomon

**Affiliations:** Research and Training Department, Amanuel Mental Specialized Hospital, PO box: 1971, Addis Ababa, Ethiopia

## Abstract

**Background:**

About 25–60% of the homeless population is reported to have some form of mental disorder. To our knowledge, there are no studies aimed at the screening, diagnosis, treatment, care, rehabilitation, and support of homeless people with mental, neurologic, and substance use (MNS) disorders in general in Ethiopia. This is the first study of its kind in Africa which was aimed at screening, diagnosis, care, treatment, rehabilitation, and support of homeless individuals with possible MNS disorder.

**Methods:**

Community-based survey was conducted from January to March 2015. Homeless people who had overt and observable psychopathology and positive for screening instruments (SRQ20, ASSIST, and PSQ) were involved in the survey and further assessed for possible diagnosis by structured clinical interview for DSM-IV diagnoses and international diagnostic criteria for seizure disorders for possible involvement in care, treatment, rehabilitation services, support, and training. The Statistical Program for Social Science (SPSS version 20) was used for data entry, clearance, and analyses.

**Results:**

A total of 456 homeless people were involved in the survey. Majority of the participants were male (*n* *=* 402; 88.16%). Most of the homeless participants had migrated into Addis Ababa from elsewhere in Ethiopia and Eritrea (62.50%). Mental, neurologic, and substance use disorders resulted to be common problems in the study participants (92.11%; *n* *=* 420). Most of the participants with mental, neurologic, and substance use disorders (85.29%; *n* *=* 354) had psychotic disorders. Most of those with psychosis had schizophrenia (77.40%; *n* *=* 274). Almost all of the participants had a history of substance use (93.20%; *n* *=* 425) and about one in ten individuals had substance use disorders (10.54%; *n* *=* 48). Most of the participants with substance use disorder had comorbid other mental and neurologic disorders (83.33%; *n* *=* 40).

**Conclusion and recommendation:**

Mental, neurologic, and substance use disorders are common (92.11%) among street homeless people in Ethiopia. The development of centers for care, treatment, rehabilitation, and support of homeless people with mental, neurologic, and substance use disorders is warranted. In addition, it is necessary to improve the accessibility of mental health services and promote better integration between mental and primary health care services, as a means to offer a better general care and to possibly prevent homelessness among mentally ill.

## Background

Homelessness problem is most often caused by multiple and interrelated individual structural depreciation. It leads to maladjustment and creates problems related to issues of social, economic, health, and starvation [1]. Homelessness is defined in various ways; in this study, homelessness indicates only those sleeping in designated shelters or public spaces. Broadly the term can include people living in marginal accommodation, as well as roofless people. Some studies include the former, while in others the definition includes only those sleeping in designated shelters or public spaces. The features of a definition of homelessness that are useful in any locality, however, are the lack of appropriate housing and the social marginalization of the individual [2, 3].

Majority of those people sleeping in designated shelters or public spaces have nationalities of the country of residence but some of them may be immigrants or nationality of other countries. The population of homeless persons is very diverse: it includes representatives from all ethnic groups, the young and the old, women and men, single persons and families, people from both urban and rural environments, and people with physical and/or mental problems [4, 5]. Most of the homeless individuals are between 31 and 50 years of age, are unmarried, and are unemployed [6–10].

Homeless people are more likely than the general population to have mental, neurologic, and substance use disorders. Evidence from different countries indicated that most of the homeless people suffer from mental illnesses such as depression, schizophrenia, substance abuse, psychotic disorders, and personality disorders. The prevalence of these disorders among homeless populations varies from country to country, and the precise cultural, national, psychosocial, and neurobiological determinants of these differences remain unclear. However, trends in mental disorders, homelessness, drug abuse, and crime suggest that Western industrial societies are becoming increasingly harmful to psychological and social well-being [11, 12].

According to different studies, 25–50% of the homeless population is reported to have some form of mental disorder [13–15] in high-income countries. This rises to about 60% among those who are street homeless. Severe mental disorders (SMD) and addictive or substance use disorders are the most common conditions among homeless individuals. In a meta-analysis of 29 studies conducted from 1979 to 2005, alcohol and drug dependence are the most common problems in homeless people with a pooled prevalence estimate of 38 and 24%, respectively, followed by psychosis with the pooled prevalence estimate of 13% [16]. Other studies indicated that 20–25% of the homeless population in the United States suffers from some form of severe mental illness. In comparison, only 6% of Americans are severely mentally ill [17]. In addition, in United States, mental illness was the third largest cause of homelessness [17].

As compared to general populations, homeless persons suffer from a high prevalence of physical disease, mental illness, and substance abuse [18–28]. Homelessness is associated with an increased risk of infections such as tuberculosis and human immunodeficiency virus (HIV) disease [29–34]. Among the homeless, access to health care is often suboptimal [35–39]. Homeless persons also experience severe poverty and often come from disadvantaged minority communities, factors that are independently associated with poor health [40–45].

Evidences from different studies have shown that the presence of mental disorders homeless people increases likelihood that the person spends longer periods as homeless [46], increased risk of mortality from general medical causes, suicide [47–49] and drug-related causes [50] and also increases vulnerability of the homeless person, including violent victimization [51] and criminality [52–54].

Evidence from studies in China [55], Nigeria [56], and Ethiopia [57] in people with schizophrenia revealed prevalence estimates of homelessness, i.e., 7.8, 4, and 7%, respectively. To our knowledge, there are no studies aimed at the screening, diagnosis, treatment, care, rehabilitation, and support of homeless people with mental, neurologic, and substance use (MNS) disorders in general in Ethiopia. This is the first study of its kind in Ethiopia, probably in Africa which aimed at screening, diagnosis, care, treatment, rehabilitation, and support of homeless individuals with possible MNS disorder. Furthermore, this study was aimed at creating continued rehabilitation, training, and other support for homeless people with MNS disorder in Ethiopia.

## Methods

### Study setting and design

Community-based survey was conducted from January to March 2015, at Addis Ababa, Ethiopia. Addis Ababa is the capital and largest city of Ethiopia. It has a population of 3,384,569 according to the 2015 population census, with annual growth rate of 3.8%. This number has been increased from the originally published 2,738,248 figure and appears to be still largely underestimated [58, 59]. As a chartered city, Addis Ababa has the status of both a city and a state. It is where the African Union is and its predecessor the OAU was based. It also hosts the headquarters of the United Nations Economic Commission for Africa (ECA) and numerous other continental and international organizations. Addis Ababa is therefore often referred to as “the political capital of Africa” due to its historical, diplomatic, and political significance for the continent [60]. In Ethiopia, particularly in major cities, homelessness is a manifest problem. In Addis Ababa, for example, the city administration estimates the number of homeless individuals to be around 50,000. A total of 456 homeless individuals were participated in the survey.

### Study population

A total of 456 homeless people (homelessness) equated with street homelessness (rooflessness) were included in the survey. All homeless people are included from Addis Ababa, Ethiopia.

### Sampling procedures

Study participants were included using multistage cluster sampling technique. We randomly selected 30 wards (kebeles) from a total of 98 in Addis Ababa, and everyone within the chosen wards (kebeles) who fulfilled the inclusion criteria was sampled.

### Inclusion and exclusion criteria

Homeless people who had overt and observable psychopathology and positive for screening instruments (SRQ20, ASSIST and PSQ) were involved in the survey and further assessed for possible diagnosis by structured clinical interview for DSM-IV diagnoses and international diagnostic criteria for seizure disorders for possible involvement for care, treatment, rehabilitation service, support, and trainings.

### Data collection, processing, and analyses

Data were collected by trained psychiatry professionals. The SRQ20, ASSIST, and PSQ were used for screening for possible problems, and Structured Clinical Interview of DSM-IV (SCID) was administered by psychiatry professionals and used to assess mental, neurologic, and substance use disorders. The Statistical Program for Social Science (SPSS version 20) was used for data entry, clearance, and analyses. Sociodemographic and clinical factors (diagnosis, history of alcohol, cannabis, nicotine, and khat abuse or dependence) were analyzed and reported using words, tables, and charts.

### Ethical consideration

The survey was led by Amanuel Mental Specialized Hospital and was collaborative work with the Federal Ministry of Health of Ethiopia and Addis Ababa Health Bureau. Ethical approval was obtained from Amanuel Mental Specialized Hospital and Addis Ababa Health bureau Research Ethics Committee (REC). Written informed consent was obtained from each study participant and they were informed about their rights to interrupt the interview at any time. Confidentiality was maintained at all levels of the survey. The survey or research team facilitated admission, in collaboration with the district administration and Amanuel Mental Specialized Hospital, for those who had possible mental, neurologic, and substance use disorders and considered at immediate risk, including admission to a rehabilitation unit. All homeless people with possible mental, neurologic, and substance use disorders were evaluated by a specialist for diagnosis and further management and most of them were treated in inpatient unit at primary-, secondary-, and tertiary-level health institutions for 3–6 months.

## Results

### Sociodemographic characteristics of participants

A total of 456 homeless people were involved in the survey. Majority of the participants were male (*n* *=* 402; 88.16%). Most of the homeless participants had migrated into Addis Ababa from elsewhere in Ethiopia and Eritrea (62.50%). Most homeless participants were chronically homeless, with over three-fourths (79.61%) having been homeless for 2 years or longer; only 20.39% of participants were homeless for less than 2 years (Table 1).

**Table 1:** Sociodemographic characteristics of homeless people (*n* *=* 456), Addis Ababa, Ethiopia, 2015

Variable	Frequency	%
Gender		
Female	54	11.84
Male	402	88.16
Regions (place of birth/residence before homelessness)		
Addis Ababa	171	37.50
Oromia	79	17.32
Amhara	83	18.20
South Ethiopia	64	14.04
Tigray	10	2.20
Diredawa	6	1.32
Harar	8	1.76
Somali	2	0.44
Eritrea	8	1.76
Others	25	5.46
Duration of homelessness		
< 6 months	27	5.92
6–12 months	30	6.58
1–2 years	36	7.89
2–5 years	112	24.56
> 5 years	251	55.05

### Magnitude and types of specific disorders among homeless people with mental, neurologic, and substance use disorders

In general, a majority of the participants included in the survey had some form of MNS disorder (92.11%; *n* *=* 420), and more than three-fourths of cases (85.29%; *n* *=* 354) were psychotic disorders. Most of those with psychosis had schizophrenia (77.40%; *n* *=* 274). Almost all of the participants had a history of substance use (93.20%; *n* *=* 425) and about one in ten individuals had substance use disorders (10.54%; *n* *=* 48). Most of the substance use disorder was comorbid with other mental and neurologic disorders (83.33%; *n* *=* 40) (Table 2).

**Table 2:** Prevalence of mental, neurologic, and substance use disorders among homeless participant, Addis Ababa, Ethiopia (*n* *=* 456) Addis Ababa, Ethiopia, 2015

Type of mental disorders	Frequency	%
Schizophrenia	274	60.09
Psychotic disorders NOS	45	9.87
Major depressive disorders with psychotic features	23	5.04
Substance-induced psychotic disorders	31	6.79
Major depressive disorders	21	4.61
Brief psychotic disorders	4	0.88
General anxiety disorders	4	0.88
Catatonia (unspecified)	1	0.21
Substance use history	425	93.20
Substance use disorders	48	10.53
Epilepsy	8	1.75
Comorbid mental and substance use disorders	40	83.33
No illness	36	7.89
Overall mental neurologic and substance use disorders	420	92.11

## Discussion

The current survey revealed that a significant portion of homeless study participants had mental, neurologic, and substance use disorders and the high burden of mental, neurologic, and substance use disorders among street homeless in Ethiopia. To our knowledge, there are no studies aimed at screening, diagnosis, treatment care, rehabilitation, and establishment of continued support and networks for homeless people with MNS disorder in general in Ethiopia.

This study revealed that the homeless people have very diverse sociodemographic characteristics in Ethiopia; this was comparable to that seen in homeless people in high-income country settings [4, 5]. In this survey majority, the participants had some form of MNS disorder (92.11%; *n* *=* 420), and more than three-fourths of cases (85.29%; *n* *=* 354) had psychotic disorders. Most of those with psychosis had schizophrenia (77.40%; *n* *=* 274). These findings were higher than the findings of Brazil (49%) [61] and other high-income countries (60%) [13]. The possible reason for the higher rate of mental disorder in our survey may be because in our study those who had overt and observable psychopathology and positive by screening instruments were involved, who are known to have higher rates of mental, neurologic, and substance use disorders.

The findings of the current study revealed that most of those homeless people with psychosis had schizophrenia (77.40%; *n* *=* 274). This finding was higher than the study done in high-income countries which revealed 20–25% of the homeless population in the United States suffers from some form of severe mental illness including schizophrenia [17]. The possible reason for the higher rate of schizophrenia in our survey may be because in our study those who had overt and observable psychopathology and positive by screening instruments were involved, who are known to have higher rates of mental, neurologic, and substance use disorders.

In addition, we found that one in ten individuals had substance use disorders (10.54%; *n* *=* 48). These results are lower than a report from a meta-analysis of 29 studies conducted from 1979 to 2005, which revealed pooled prevalence estimate alcohol and drug dependence, i.e, 38 and 24%, respectively, among homeless individuals [16]. The possible reason for this difference might be due to the instrument they used, socioeconomic and cultural difference, and the sample included in the study.

### Limitation of the study

In our study, those who had overt and observable psychopathology and positive by screening instruments were involved, who are known to have higher rates of mental, neurologic and substance use disorders.

### Conclusion and recommendations

Mental, neurologic, and substance use disorders resulted to be common problems in the study participants (92.11%; *n* *=* 420). Most of the participants with mental, neurologic, and substance use disorders (85.29%; *n* *=* 354) had psychotic disorders. Most of those with psychosis had schizophrenia (77.40%; *n* *=* 274). Almost all of the participants had a history of substance use (93.20%; *n* *=* 425) and about one in ten individuals had substance use disorders (10.54%; *n* *=* 48). In the current study, it was found that comorbid substance use disorder with other mental and neurologic disorders was a more frequent phenomenon among homeless people and that majority of those who have mental and neurologic disorders have co-occurring substance use disorders as compared to those who have no mental and neurologic disorders. Our findings indicate that attention needs to be given to screen and assess mental, neurologic, and substance use disorders in homeless people. The development of centers for care, treatment, rehabilitation, and support of homeless people with mental, neurologic, and substance use disorders is warranted. In addition, it is necessary to improve the accessibility of mental health services and promote better integration between mental and primary health care services, as a means to offer a better general care and to possibly prevent homelessness among mentally ill.
